# Classification of Granite Soils and Prediction of Soil Water Content Using Hyperspectral Visible and Near-Infrared Imaging

**DOI:** 10.3390/s20061611

**Published:** 2020-03-13

**Authors:** Hwan-Hui Lim, Enok Cheon, Deuk-Hwan Lee, Jun-Seo Jeon, Seung-Rae Lee

**Affiliations:** 1 Dept. of Civil and Environmental Engineering, Korea Advanced Institute of Science and Technology, Daejeon 34141, Korea; hwanhui@kaist.ac.kr (H.-H.L.); enokjun@kaist.ac.kr (E.C.); deukhwan@kaist.ac.kr (D.-H.L.); 2 Building Safety Research Center & Seismic Safety Research Center, Korea Institute of Civil Engineering and Building Technology, Daejeon 34141, Korea; junseojeon@kict.re.kr

**Keywords:** granite soils, water content, hyperspectral camera, visible and near-infrared, soil water characteristic curve, artificial neural network

## Abstract

Soil water content is one of the most important physical indicators of landslide hazards. Therefore, quickly and non-destructively classifying soils and determining or predicting water content are essential tasks for the detection of landslide hazards. We investigated hyperspectral information in the visible and near-infrared regions (400–1000 nm) of 162 granite soil samples collected from Seoul (Republic of Korea). First, effective wavelengths were extracted from pre-processed spectral data using the successive projection algorithm to develop a classification model. A gray-level co-occurrence matrix was employed to extract textural variables, and a support vector machine was used to establish calibration models and the prediction model. The results show that an optimal correct classification rate of 89.8% could be achieved by combining data sets of effective wavelengths and texture features for modeling. Using the developed classification model, an artificial neural network (ANN) model for the prediction of soil water content was constructed. The input parameter was composed of Munsell soil color, area of reflectance (near-infrared), and dry unit weight. The accuracy in water content prediction of the developed ANN model was verified by a coefficient of determination and mean absolute percentage error of 0.91 and 10.1%, respectively.

## 1. Introduction

Soil type and water content affect the physical and chemical properties of soil, and changes in soil properties can lead to landslides or debris-flows [1]. The classification of soil types and the prediction of soil water content are crucial for monitoring landslides and debris-flows [2,3]. Therefore, in order to effectively detect landslide hazards, it is essential to quickly and non-destructively classify soil and predicting water content variations arising from the infiltration of rainfall.

Soils have various compositions with different chemical and physical properties [4]. Soil color can provide information on soil formation history [5] and is a comprehensive indicator of the chemical composition and physical characteristics of soils. Considering that a significant amount of soil information can be effectively obtained by interpreting the color of soil, methods based on soil color are the most common form of soil classification and qualitative detection [6]. Most soils are shades of black, brown, red, yellow, and white [5]. In Korea, representative forest soils are classified as brown, yellow, and red forest soils [7]. Therefore, in this study, classification models were developed for these three colors. 

For most types of slope failure, soil water content plays a critical role as increased pore water pressure reduces soil strength and increases stress [8]. Accordingly, soil water content measurement is a subject that has been studied over several decades. A previous study proposed a measurement method for soil water content using the chemical reaction of calcium carbide [9]. In addition, microwave, frying pan, and radiometric measurement methods were applied to determine the water content of sites [10]. However, all of the aforementioned methods are limited, as water content can only be measured for relatively small areas. To predict disasters such as landslides and debris-flows, it is necessary to measure variations in soil water content over large areas. Therefore, new technology needs to be developed for this purpose. In this study, a model for predicting water content variations in soils was developed using hyperspectral imaging to overcome existing limitations.

Since the early 2000s, near-infrared (NIR) spectroscopy has been widely employed as a useful tool for the analysis of soil properties [11]. NIR spectroscopy can be used to evaluate the properties of soil that are not disturbed by light [12,13]. Using these spectral characteristics, hyperspectral imaging technology has been studied in various fields. In the food industry, hyperspectral imaging technology has been used for the identification of defects [14]. Valenzuela et al. [15] used visual and infrared (VIR) hyperspectral imaging to determine the firmness and solid content of blueberries. In medical diagnosis, Mitra et al. [16] scanned the biliary structure using both fluorescence and hyperspectral imaging for the classification of different tissues and identification of the biliary anatomy. Hyperspectral imaging technology has also been used for applications in precision agriculture. Zhang et al. [17] used hyperspectral imaging technology and deep learning for feature extraction as well as for crop monitoring to detect diseases, water stress, nutrients, and insect attacks. In this study, we developed a soil classification and prediction method for soil water content variation that works over large areas using machine learning methods and hyperspectral imaging in the visible and near-infrared regions (VNIR, 400–1000 nm). Among the machine learning methods, the artificial neural network (ANN) model was selected, and the hyperspectral imaging analysis provided the input parameters for the ANN model.

The hyperspectral imaging analysis was conducted to confirm the change in reflectance according to soil color. The successive projections algorithm (SPA) was applied to select the effective wavelengths among the hyperspectral data. SPA is a commonly used algorithm for wavelength selection in multivariate calibration and classification [18]. The algorithm reduces multiple collinearity and redundancy in the full wavelength and selects only a few wavelengths with useful information, which helps to reduce the amount of data, simplifying calculations and allowing for the development of simple and powerful models [19,20]. A combination of image texture features and hyperspectral analysis was shown to increase the accuracy of soil color classification [21]. In the image classification model, the most common and effective method for texture feature analysis is the gray level co-occurrence matrix (GLCM) [22,23]. Textural features based on gray-tone spatial dependencies have general applicability in image classification. Textural features contain information regarding image texture characteristics such as homogeneity, gray-tone linear dependencies, contrast, number, the nature of the boundaries present, and the complexity of the image [23].

In this study, (1) soil color was classified using hyperspectral image analysis and texture features, and (2) an ANN model was developed to predict variations in soil water content. The developed methodologies and the results of this study can be used to analyze water content variations of soil through remote sensing.

## 2. Materials and Methods

### 2.1. Study Area Descriptions

**Figure 1:**
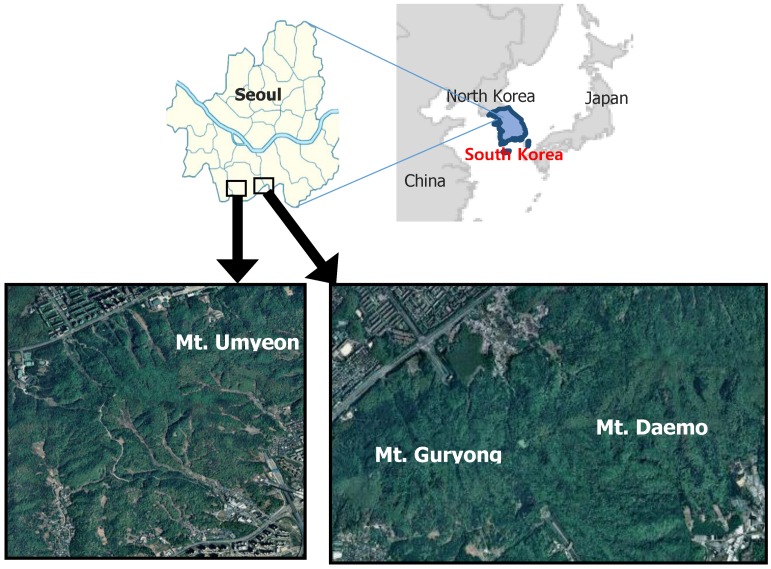
Location map showing the corresponding study area sample locations.

The study areas are located in the southern part of Seoul (Mt. Umyeon: 37.45° N, 126.9° E; Mt. Guryong: 37.47° N, 127.06° E; Mt. Daemo: 37.48° N, 127.08° E), as shown in Figure 1. The study areas consist of granitic gneiss.

**Figure 2:**
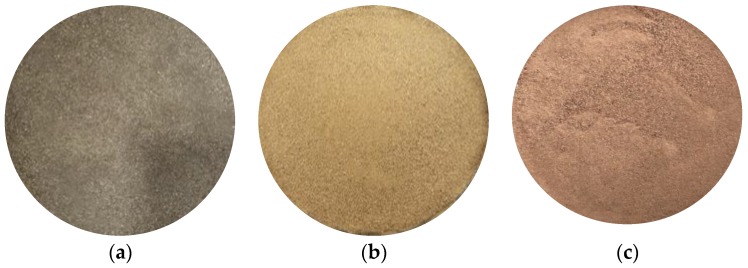
Red, green, and blue (RGB) images of (**a**) Brown, (**b**) Yellow, and (**c**) Red soils after No. 40 sieve analysis.

A total of 162 granite soil samples were sampled from the study areas. In this study, granite soil samples were categorized into three types based on color. The details are shown in Table 1. Figure 2 shows the red, green, and blue (RGB) images of the three granite soil samples. The soil samples were collected within a depth of 30 cm from the surface. First, a sieve analysis was performed using soil that passed through a No. 40 sieve (0.425 mm). The soils were also dried at 110 ℃ in an oven for 24 hours.

### 2.2. Hyperspectral Camera System

**Figure 3:**
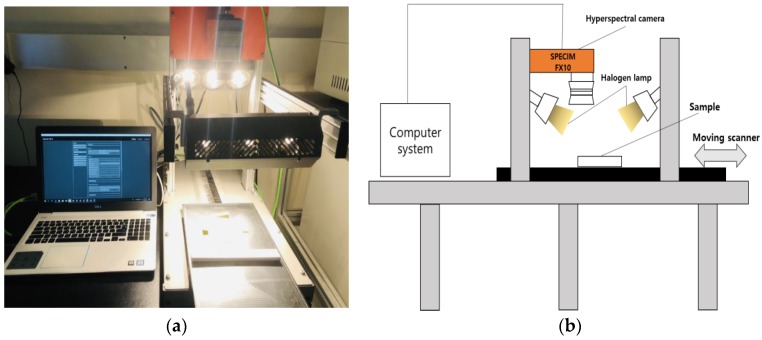
Hyperspectral camera system: (**a**) hyperspectral camera in the laboratory and (**b**) schematic diagram of the hyperspectral camera.

The hyperspectral camera system was composed of a hyperspectral camera, a complementary metal-oxide semiconductor (CMOS) sensor, six 150W halogen lamps, and a 40×20 Lab-scanner (Spectral Imaging Ltd., Oulu, Finland, Figure 3a). The hyperspectral camera used in this study was a SPECIM FX10 (Spectral Imaging Ltd.) which uses the pushbroom scanning method. The main characteristics of the hyperspectral camera are shown in Table 2. The Lumo Recorder software (Middleton Spectral Vision, Middleton, WI, USA) provided scanning speed (computer numerical control (CNC) USB controller), and a hyper-cube data recorder provided exposure time, binning mode, wavelength range, and image acquisition [24]. The hyperspectral cameras were placed in a dark room to minimize errors. The soil samples were placed into Schale dishes with a diameter of 60 mm. The Schale dishes were placed on the Lab-scanner for image acquisition. Hyperspectral images can be acquired with spectral and image information. To acquire clear and error-free hyperspectral images, the scanning speed of the Lab-scanner, the exposure time of the camera, and the height between the lens of the camera and the sample were set to 13.7 mm/s, 29.22 ms, and 30.0 cm, respectively. The hyperspectral images were analyzed with ENVI Classdic5.5 (ITT, Visual Information Solutions, Boulder, CO, USA) [25]. A schematic diagram of the hyperspectral camera is shown in Figure 3b.

### 2.3. Image Correction

Hyperspectral images were obtained using the line scanning technique of the hyperspectral camera system. Each sample was placed on a slider table and scanned line by line to obtain an initial hyperspectral image. After capturing the hyperspectral image, a dark reference and a white reference were taken. The normalization process removed the noise value from the hyperspectral image and converted it to a relative value with 100% reflectance of the white reference. The white reference was obtained from a Teflon whiteboard with 99% reflectivity, and the dark reference was obtained by turning off the light source and completely covering the camera lens with a cap. The reflectance (%) of the sample obtained based on the white reference was calculated using the following equation:

(1) $$\mathrm{Reflectance}=\frac{Raw^{t1}-Dark^{t1}}{White^{t2}-Dark^{t2}}\times \frac{t2}{t1}$$

where the *Raw reflectance* is the reference measured on an actual object, *Dark* is the dark reference, *White* is the white reference, *t*1 is the integration time in a white reference, and *t*2 the integration time in a dark reference [26].

### 2.4. Region of Interest (ROI) Selection 

**Figure 4:**
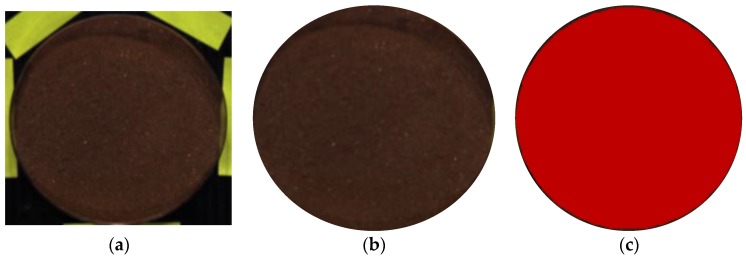
Soil image showing the “region of interest (ROI)” representing the average selected pixels: (**a**) hyperspectral image (specimen from Mt. Guryong), (**b**) remaining image, and (**c**) the region of interest in the granite soil.

First, the background parts of the hyperspectral images of each soil sample, except the soil, were removed (Figure 4b). Afterward, only the remaining soil was selected as the region of interest (ROI), as shown in Figure 4c. The ROI can be manually selected using geometric shapes, such as a rectangle, circle, or polyline, drawn using the ROI tool in ENVI Classic5.5 [25]. When the ROI is selected, the background is removed automatically. The reflectance values of all pixels in the ROI were averaged to generate a single average reflectance value. To reduce spectral noise and error, the beginning and end of the spectrum were removed, and only wavelengths within 400–1000 nm (204 bands) were used. The same procedure was repeated for all ROI images of the 162 soil samples.

### 2.5. Spectral Feature and Image Texture Feature Extraction

#### 2.5.1. Spectral Pre-Processing

In this study, two steps were used to extract spectral features. Firstly, spectra were pre-processed through three pre-processing methods, after which effective wavelengths were selected using SPA. 

The spectral curve of the sample may include a certain amount of noise caused by physical and chemical factors and the data acquisition equipment. This noise can reduce the signal-to-noise ratio (SNR) and resolution of the signal, reducing the accuracy and precision of the calibration model. Various pre-processing methods have been proposed for effective pretreatment. Selecting the appropriate pre-processing method according to the characteristics of the data and the purpose of the experiment helps to improve the performance of the regression model [27]. 

The pre-processing methods can be largely divided into scatter correction and spectral derivation. Scatter correction methods correct the effects of atmospheric scattering and include Multiplicative Scatter Correction (MSC) and Standard Normal Variate (SNV). MSC performs calibrations assuming the average value at each wavelength as the ideal value, and SNV normalizes each spectral curve to the standard deviation of the entire spectral curve to eliminate the effects of scattering [28]. Spectral derivation methods include first derivative and second derivative methods [29]. In this study, the first and second derivatives were used with the exception of the scattering correction method, as the test was at a laboratory scale.

#### 2.5.2. Selection of Effective Wavelength

In hyperspectral images, bands may be too numerous, depending on the wavelength. Therefore, an effective wavelength should be selected to reduce the calculation load and eliminate redundant information. SPA is considered an effective wavelength selection method that can minimize multicollinearity among variables. Therefore, SPA was used for effective wavelength selection to improve the prediction accuracy and calculation speed of the classification models [30]. This procedure was conducted in the MATLAB software (The MathWorks 2014, Inc., Natick, MA, USA) [31].

#### 2.5.3. Image Texture Feature Extraction

Texture features were extracted using a gray-level co-occurrence matrix (GLCM). A GLCM creates a matrix from a particular image and calculates how often a pixel with gray-level (grayscale intensity) value *i* occurs horizontally adjacent to a pixel with value *j*. GLCMs involve several properties and parameters for texture feature extraction [32]:

(1) Contrast: Returns a measure of the intensity contrast between a pixel and its neighbor over the entire image:
  (2) $$\mathrm{Con}=\sum_{I=0}^{N-1}\sum_{J=O}^{N-1}{\left(i-j\right)}^{2}M\left(i,j\right)$$
(2) Correlation: Returns a measure of how correlated a pixel is to its neighbor over the entire image:
  (3) $$C=\frac{\sum_{I=0}^{N-1}\sum_{J=0}^{N-1}ijM\left(i,j\right)-I_{2}^{2}}{I_{3}}$$
(3) Energy: Returns the sum of the squared elements in the GLCM:
  (4) $$E=\sqrt{\sum_{i=0}^{N-1}\sum_{j=0}^{N-1}M^{2}\left(i,j\right)}$$
(4) Homogeneity: Returns a value that measures the closeness of the distribution of elements in the GLCM to the GLCM diagonal:
  (5) $$H=\sum_{i=0}^{N-1}\sum_{j=0}^{N-1}\frac{M\left(i,j\right)}{1+\left|i-j\right|}$$

The GLCM textures included contrast, correlation, energy, and homogeneity extracted from four directions (0°, 45°, 90°, and 135°), and a distance of one pixel was applied [33]. A total of 204 gray-scale images were obtained, and when the texture features were calculated for all grayscale images, large amounts of duplicate information were generated. Therefore, in this study, the texture features were extracted only from gray-scale images for each effective wavelength.

#### 2.5.4. Classification Models and Regression Analysis

A Support Vector Machine (SVM) was used to establish classification models based on the texture features and effective wavelength. SVMs are used to handle classical two-class pattern recognition problems. An SVM is a supervised machine learning algorithm that can be used for both classification and regression tasks [27], but is mainly used in classification models. The 162 hyperspectral images of granite soils were randomly assigned to the training set and testing set with a 7:3 ratio, which is equivalent to a total of 38 calibration samples and 16 validation samples for brown soil samples; 32 calibration samples and 14 validation samples for yellow soil samples; and 29 calibration samples and 13 validation samples for red soil samples. First, the effective wavelength was selected using SPA, after which the texture feature corresponding to the effective wavelength was extracted using the GLCM. Finally, a classification model was constructed using the SVM algorithm.

#### 2.5.5. Prediction of Soil Water Content Variation

As in the case of the classification model, the hyperspectral data of the 162 granite soil samples were divided into training and testing sets with a 7:3 ratio. An ANN is a powerful computational technique used for the capturing and modeling of nonlinear and complex relationships of variables embedded in a small set of data [34]. In addition, an ANN is a type of non-linear processing system that is perfectly appropriate for a widespread domain of applications [35]. ANNs utilize connected artificial neurons, and its inherent behaviors can be explained by training the input parameters using the neurons, which results in nonlinear mapping [36]. Such networks can achieve a high level of accuracy without requiring large amounts of training data. The accuracy of prediction seems to be more dependent on the number of layers in the neural network than the number of neurons [37]. For these reasons, among the various machine learning methods that use neural networks, this study utilized an ANN for the prediction of the soil water content. ANNs are structured with three layers: an input layer, a hidden layer, and an output layer. Three parameters were selected for the ANN input layer, and soil water content was selected for the ANN output layer. One of the input parameters was soil color using the Munsell color chart, which was converted from the RGB values. The most common method of determining soil color is through comparison with the Munsell color chart [38,39]. Another parameter was the area of reflectance (NIR). In the VNIR region, the area of reflectance (NIR, 800–1000 nm) is suggested as having a strong correlation with soil water content [40]. The final parameter was dry unit weight, which considers the physical properties of the soil. Moreover, dry unit weight also has strong correlations with water content [41]. To construct an optimal ANN structure, the number of neurons in the hidden layers was changed from one to eight, and several combinations of transfer functions were considered (Table 3).

### 2.6. Overall Developed Workflow 

**Figure 5:**
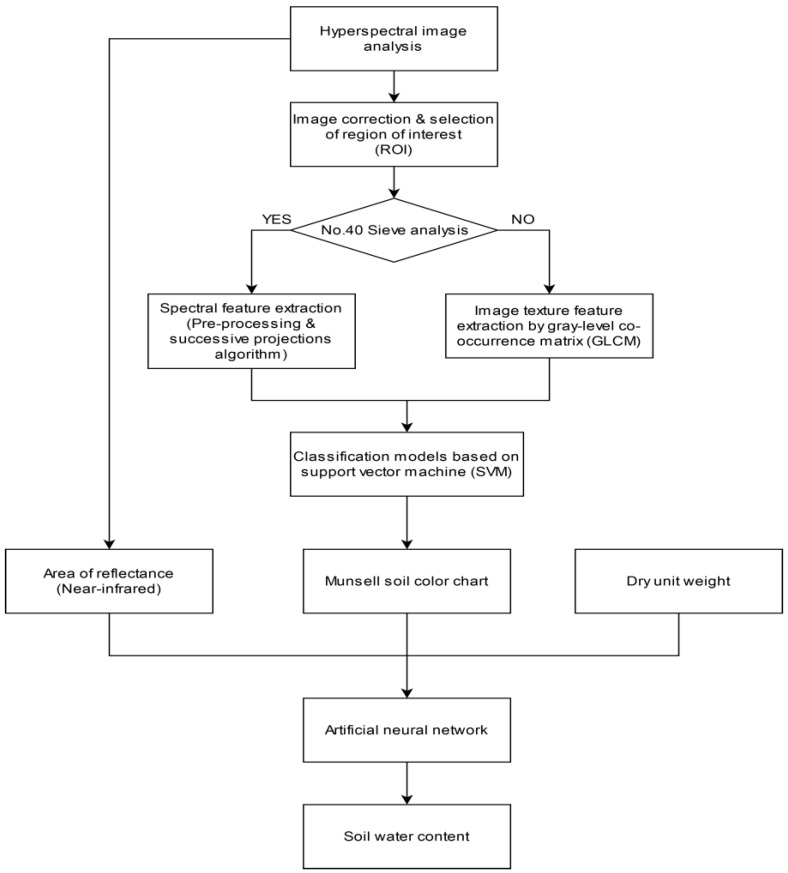
Overall flowchart showing the sequence of steps involved in the procedure of soil classification and the artificial neural network (ANN) for the prediction of soil water content.

The overall flowchart of the study procedure is shown in Figure 5. Hyperspectral image analysis was performed on a total of 162 granite weathered soil samples. After image correction and ROI selection, the effective wavelength was selected through pre-processing and the use of SPA in the spectral feature extraction step. Next, texture features were extracted using a GLCM in the image texture feature step. Based on the GLCM and selected effective wavelength, an SVM-based classification model was developed to determine the color of the soil sample, which is represented as a Munsell soil color. The 162 hyperspectral images of the granite soils were randomly assigned to the training set and testing set with a 7:3 ratio. The ratios of the calibration validation samples for brown soils, yellow soils, and red soils were 38:16, 32:14, and 29:13, respectively. An ANN model was developed to predict the variation of soil water content using the Munsell soil color, the dry unit weight of the soil, and the area of reflectance (NIR) obtained from the hyperspectral image analysis.

## 3. Results and Discussion

### 3.1. Comparison of Pre-Processing Methods

In this study, the first and second derivatives were selected as pre-processing methods. Table 3 shows the prediction accuracies of the pre-processing methods.

As shown in Table 4, the second derivative method was more accurate than the first derivative method. Therefore, the second derivative method was selected as the pre-processing method.

### 3.2. Selection of Effective Wavelength

**Figure 6:**
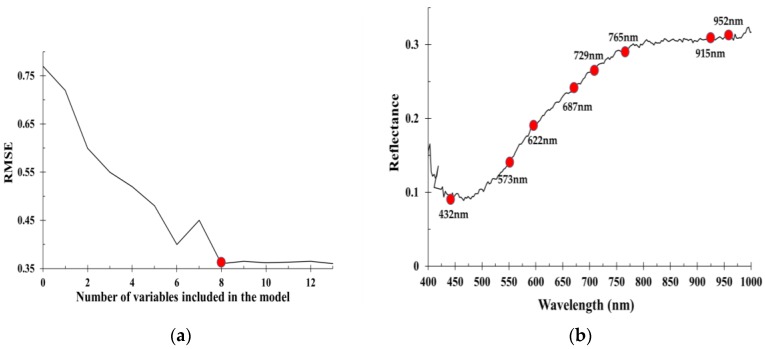
Selection of effective wavelengths using the successive projections algorithm: (**a**) Number of selected variables; (**b**) Effective wavelengths in visible and near-infrared regions.

SPA was used to select the effective wavelength from the total wavelength spectrum in VNIR. Eight variables were determined based on the minimum root mean square error of validation (RMSEV). Figure 6 shows that eight wavelengths (432, 537, 622, 687, 729, 765, 915, and 952 nm) were effectively obtained in the VNIR range (435–898 nm). In the spectrum, 432 nm shows blue (430–475 nm), 537 nm shows green (495–575 nm), 622 nm shows red (620–680 nm), 687 nm shows red-edge (680–690 nm), 729–765 nm indicates N-H stretching of the amino acids, and 915 nm and 952 nm correspond to carbohydrates and O-H stretching, respectively [42].

The selection of effective wavelengths through the previous analysis notably reduced the number of wavelengths. SVM models were subsequently constructed based on four input variables. Table 5 provides the SVM model classification results using four input variables. The first variable of the full wavelength had 204 bands with accuracies of 87.8% for the training set and 84.2% for the testing set. The second variable with effective wavelengths exhibited accuracies of 80.2% for the training set and 85.3% for the testing set. The accuracies of the third SVM model with 32 GLCM texture feature variables (four features×eight bands) were 72.3% for the training set and 69.7% for the testing set. Lastly, using both the effective wavelengths and texture features achieved accuracies of 92.3% for the training set and 89.8% for the testing set. Therefore, the effective wavelengths and texture features used in tandem were considered to be the ideal input variable for the classification of soil types as the fourth case had higher accuracies for the training and testing sets compared to the other input variables.

### 3.3. Optimal ANN Structure for the Prediction of Soil Water Content

**Figure 7:**
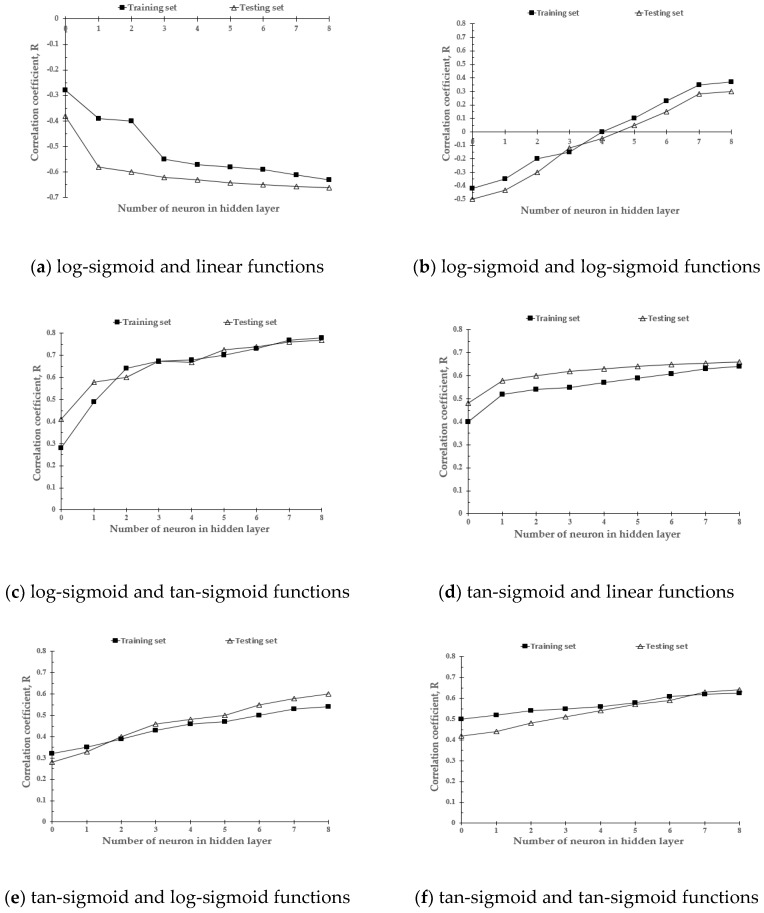
Correlation coefficients of training and testing sets: (**a**) log-sigmoid and linear functions, (**b**) log-sigmoid and log-sigmoid functions, (**c**) log-sigmoid and tan-sigmoid functions, (**d**) tan-sigmoid and linear functions, (**e**) tan-sigmoid and log-sigmoid functions, and (**f**) tan-sigmoid and tan-sigmoid functions.

**Figure 8:**
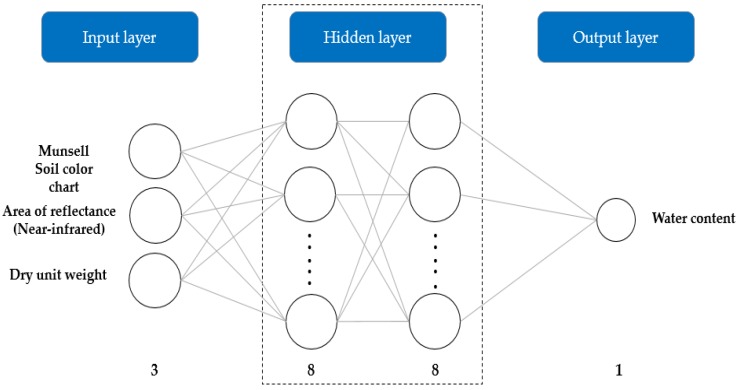
Structure of the artificial neural network (ANN) model for the estimation of soil water content.

Figure 7 shows the correlation coefficients for training and testing according to the transfer function and number of neurons in the hidden layer. The correlation coefficient ranged between −1 and 1: the closer the absolute value is to 1, the higher the predictive accuracy of the model. As previously mentioned, the structure of the ANN included three input parameters, eight neurons in the hidden layers, and one output parameter. The transfer functions were a log-sigmoid in the first hidden layer, a tan-sigmoid function in the second hidden layer, and a pure linear function in the output layer. Bayesian regularization was applied to a back-propagation neural network [43]. Such regularization reportedly minimizes the over-fitting problem with insufficient data. Figure 8 shows the structure of an optimal ANN model for the estimation of soil water content.

### 3.4. Validation of the Selected ANN Model

**Figure 9:**
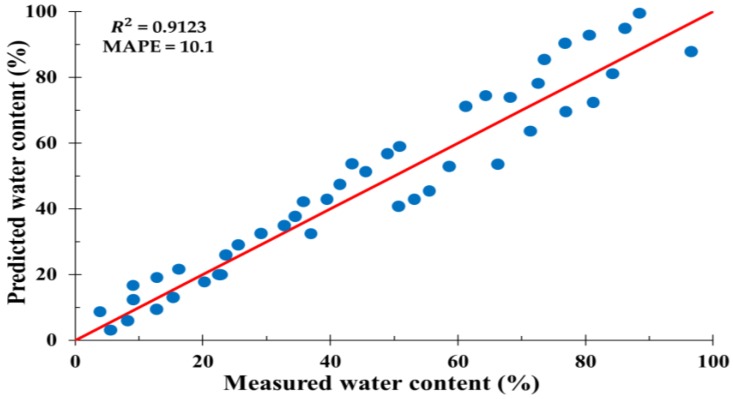
Comparison of measured water content versus water content predicted using the ANN model.

**Figure 10:**
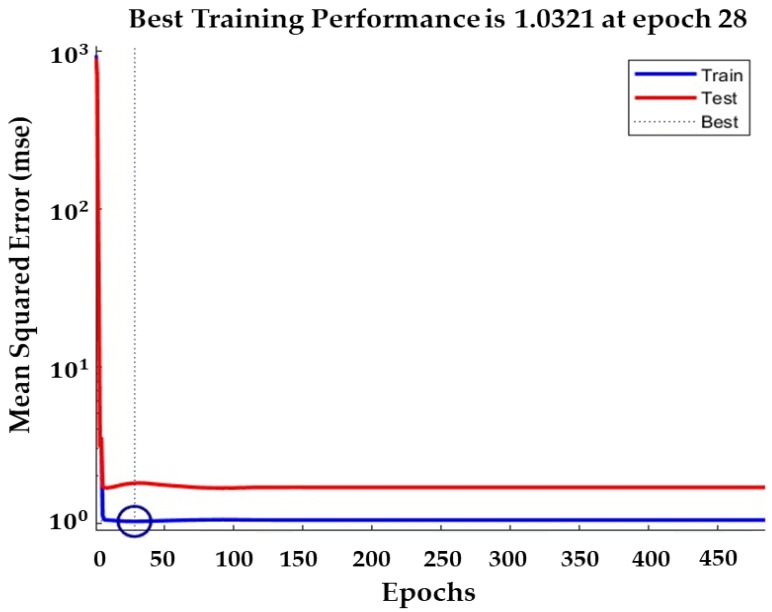
ANN convergence performance for training and testing steps.

In general, mean absolute percentage error (MAPE), root mean square error (RMSE), mean absolute error (MAE), and maximum absolute percentage error (Max-APE) are the indicators used to evaluate the goodness of fit of predictive models [44]. Among these indicators, MAPE has become increasingly popular as a performance measure in forecasting [45,46,47], as it is easy to interpret and understand in addition to being highly reliable [48]. The coefficient of determination (*R*^2^) was calculated for all data points by comparing the results predicted by the ANN model with the results obtained from laboratory tests. Higher *R*^2^ values indicate a reliable model with high predictive performance. The developed ANN model exhibited good prediction accuracy, generating an *R*^2^ value of 0.91 and MAPE of 10.1%, as shown in Figure 9. In addition, the convergence of the ANN for the data variables is described in Figure 10. The training performance of the ANN was determined to be highest at epoch 28, with a mean squared error of 1.0321. Therefore, it can be concluded that the ANN model can be successfully used to predict variations in soil water content accurately.

## 4. Conclusions

In this study, we demonstrated the potential of hyperspectral techniques for soil classification and subsequent soil water content estimations. Various pre-processing methods were applied for soil type classification, and a soil type classification model was constructed using a GLCM and SVM. In addition, we developed an ANN model that considers soil color, spectral reflectance, and dry unit weight to improve the estimation of soil water content. The research can be summarized as follows:

(1) A total of 162 granite weathered soil samples were collected from Mt. Umyeon, Mt. Guryong, and Mt. Daemo in Seoul. Hyperspectral near-infrared images were acquired in 224 bands from 400 to 1000nm. To reduce spectral noise and error, the beginning and end of the wavelength spectrum were removed and only 204 bands were used.
(2) The second derivative method was selected as the pre-processing method. The classification model produced the best results with a combination of eight effective wavelengths and GLCM-texture features of contrast, correlation, energy, and homogeneity. The testing set accuracy of the classification model was 89.8%.
(3) An optimal ANN model was developed for water content prediction. The ANN had three input parameters, eight neurons in the hidden layers, and one output parameter. The transfer functions involved were a log-sigmoid function in the first hidden layer, a tan-sigmoid function in the second hidden layer, and a pure linear function in the output layer. The developed ANN model exhibited good prediction accuracy, generating an R2 value of 0.91 and a MAPE of 10.1%. In addition, the training performance in terms of the convergence of ANN for the data variables was the highest at epoch 28 with a mean squared error of 1.0321. Therefore, it can be concluded that the ANN model can be successfully used to predict variations in soil water content accurately.

The aim of this study was to classify soil types and predict soil water content over large areas for the detection of landslide hazards, which traditionally require considerable time and human power, by implementing a simple method using hyperspectral imaging. A total of 162 granite soils (Mt. Umyeon, Mt. Guryong, and Mt. Daemo) were examined for the application of hyperspectral imaging. The results demonstrated that the developed models were capable of soil type classification and water content prediction. Presently, as there is a lack of research on the acquisition of soil properties over large areas using hyperspectral imaging, the proposed method can be used to provide basic data for such investigations. In addition, drones and the normalized difference vegetation index (NDVI) could be used to classify soil and measure water content over large areas, enabling disaster prevention. Site investigations that consider NDVI and water content will be performed to demonstrate the application of the developed method. Due to the fact that soil is exposed to various conditions depending on the weather, the proposed methods may be less accurate than conventional measurement methods. To overcome these limitations, atmospheric, and radiation correction steps will be specified, and the latest machine learning techniques will be applied in a future study.

## Figures and Tables

**Table 1:** Types of soil samples.

Type	Sample Number
Brown soils	61
Yellow soils	52
Red soils	49

**Table 2:** SPECIM FX10 main characteristics.

Parameter	Value
Spectral Range	400 nm~1000 nm
Spectral Bands	224
Spatial Sampling	1024 px
Spectral Full width at half maximum	5.5 nm
Field of view (α)	38°
Camera Signal to noise ratio (Peak)	660:1
Dimensions	150×85×71 mm
Weight	1.26 kg

**Table 3:** Combinations of transfer functions.

Number of Neurons	Combination of Transfer Functions
1–8	log sigmoid—pure linear
	log sigmoid—log sigmoid
	log sigmoid—tan sigmoid
	tan sigmoid—pure linear
	tan sigmoid—log sigmoid
	tan sigmoid—tan sigmoid

**Table 4:** Accuracy of the pre-processing methods.

Pre-Processing Method	Calibration (Support Vector Machines)	Prediction (Support Vector Machines)
First Derivative	77.8	76.9
Second Derivative	82.2	80.8

**Table 5:** Hyperspectral imaging for soil type classification with SPA and SVM.

Input	Parameters (C, g) ^1^	Training Set Accuracy	Testing Set Accuracy
Full wavelength	(53.34, 1.52)	87.8 %	84.2 %
Effective wavelengths	(38.22, 5.4)	90.2 %	85.3 %
GLCM-Texture features	(98.73, 1.22)	72.3 %	69.7 %
Effective wavelengths + GLCM-Texture features	(170.36, 5.33)	92.3 %	89.8 %

^1^ C is the optimal penalty coefficient and g is the kernel function parameter.
